# Quantitative T1 mapping and the fibrotic index in normal healthy volunteers; relationship to aging and cardiac dimensions

**DOI:** 10.1186/1532-429X-14-S1-O83

**Published:** 2012-02-01

**Authors:** Tomas G Neilan, Eri Watanabe, Otavio R Coelho-Filho, Ravi Shah, Yucheng Chen, Bobby Heydari, Ron Blankstein, Raymond Kwong, Michael Jerosch-Herold

**Affiliations:** 1 Cardiology, Brigham and Women's Hospital/Massachusetts General Hospital, Boston, MA, USA; 2 Cardiovascular Division, Brigham and Women's Hospital, Boston, MA, USA; 3 Radiology, Brigham and Women's Hospital, Boston, MA, USA

## Summary

We aimed to publish normal data for the fibrotic index in a group of healthy volunteers. In healthy volunteers, the fibrotic index has acceptable test characteristics, has a range from 0.23 to 0.33, and is associated with age, LA volume and LV mass.

## Background

Current cardiac magnetic resonance (CMR) techniques provide an accurate assessment of replacement myocardial fibrosis (MF), but are of limited value for the detection and quantification of diffuse MF. T1 mapping is a novel approach for quantification of the expansion of the extracellular matrix. However, there are little published data on the normal range of the FI and its variation with age and gender.

## Methods

Twenty seven healthy volunteers underwent a standard CMR with administration of gadolinium (Gd). T1 measurements, extending to 30 minutes post-contrast, were performed with a segmented, breath-hold Look-Locker sequence in 3 short axis slices, using 100 ms, and 50 ms for temporal resolution of one pre- and three post-contrast acquisitions, respectively. We tested the segmental, inter-slice, inter-, intra-, and test-retest characteristics of the fibrotic index, as well as the association with other cohort characteristics.

## Results

**Figure 1:**
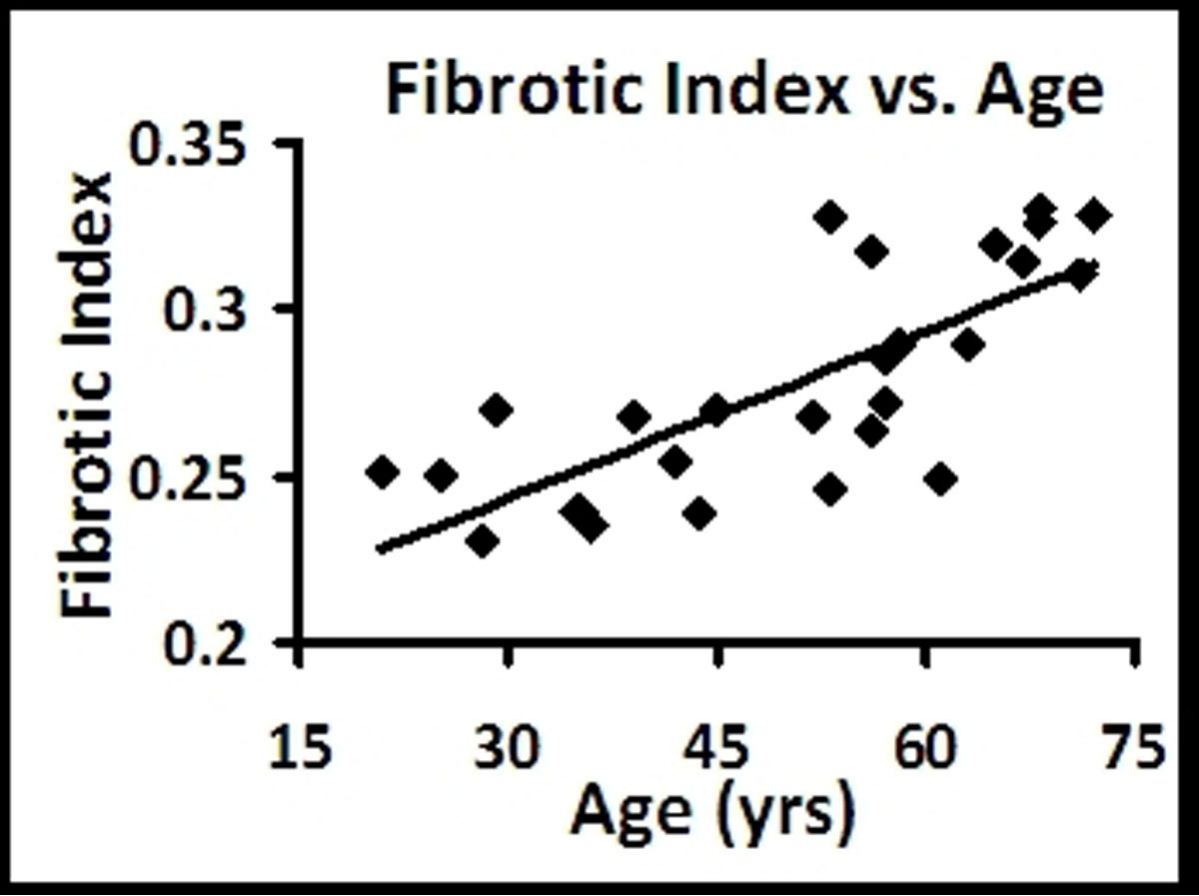


**Figure 2:**
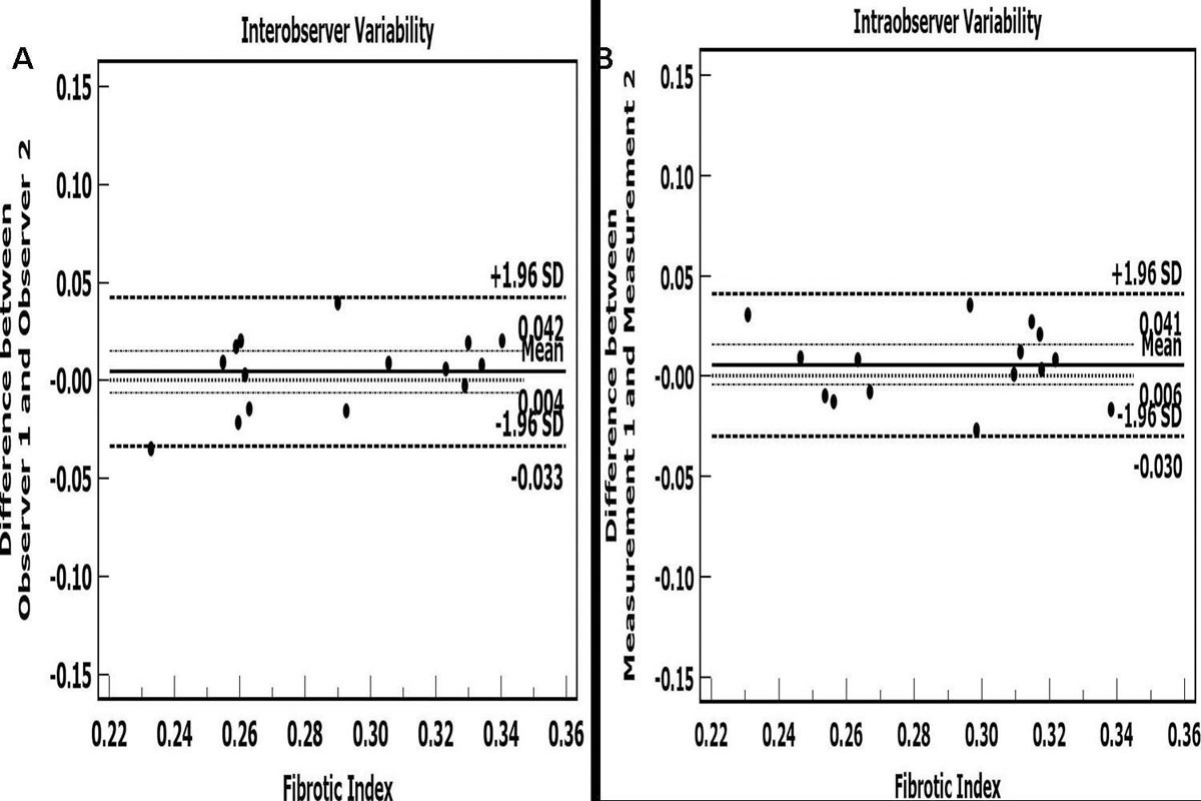


Healthy (N=27) volunteers were 52% female, ranged in age from 21 to 72, had a BMI of 27±6 kg/m2, an LVEDV of 127±28 ml, an LV mass index of 44±8 gms/m2, a max LA volume index of 33±10 ml/m2, and an EF of 64±7%. The FI averaged 0.28±0.03 (range 0.23 to 0.33), and was associated with age (r = 0.76, p < 0.001, Figure 1), maximal LA volume index (r = 0.67, p < 0.001), and indexed LV mass (r = 0.52, p < 0.01). The FI was similar in males and females (0.27±0.03 vs. 0.28±0.03, p = 0.12), however, the female cohort tended to be older than males (54 years vs. 45 years, p = 0.14). There were no differences in the FI between segments in a slice or between slices. The intra-, inter-, and test-retest characteristics of the FI were acceptable (difference 0.004, 95% CI -0.006 to 0.015, 4%, Figure 2A; difference 0.01, 95% CI -0.01 to 0.01, 5%, Figure 2B; difference 0.005, 95% CI -0.005 to 0.005, 2%).

## Conclusion

In healthy volunteers, the fibrotic index has acceptable test characteristics, has a range from 0.23 to 0.33, and is associated with age, LA volume and LV mass.

## Funding

Dr. Neilan is supported by an NIH T32 Training Grant (T32HL09430101A1).

Dr. Kwong receives salary support from a research grant from the National Institutes of Health (R01HL091157).

Dr. Jerosch-Herold is supported in part by a research grant from the National Institutes of Health (R01HL090634-01A1).

